# Hospital Meetings

**Published:** 1905-02-18

**Authors:** 


### HOSPITAL MEETINGS.

### ST. MARK'S HOSPITAL.

The annual meeting of St. Mark's Hospital for Fistula and other diseases of the rectum was held on the 9th inst., Mr. E. F. Carey presiding in the absence of the Treasurer, Mr. R. B. Martin, M.P.

In moving the adoption of the report and accounts for 1904, the Chairman drew attention to the unprecedented excess of income over expenditure, amounting, it was true, to only a small sum—£260 10s. 11d.—but one on which the hospital was to be congratulated. He urged, however, that this satisfactory state of the finances should be an inducement to make still further efforts to collect funds to establish in 1905 an even better record, and to help in recovering the very heavy deficit of 1903, when the Committee were compelled to sell capital to the extent of nearly £3,000, and to add an additional loan of £500 from the bankers. This inroad on the capital was a most grievous matter, in view of the very limited amount of invested property held by the trustees. He, therefore, desired to impress upon all friends of the hospital the very urgent need of augmenting the annual subscriptions, at present the only reliable source of income, although most inadequate to the needs of the institution. The treasurer's appeal had been very satisfactorily responded to, and accounted for an income of £1,352. There had been a slight decrease in the cost of both in and out-patients; the former had appreciably increased in numbers, while the latter remained about the same, and he felt that the most cordial thanks were due to the surgical staff, without whose co-operation these excellent results would have been impossible. In accordance with resolutions embodied in last year's report, out-patients had been requested to contribute, by small payments, for drugs and dressings, towards the expenses of the hospital, and this rule had been very cheerfully accepted by them. From this source £113 18s. had been collected. The two paying wards opened in March also showed a return of £154 16s., and the innovation seemed to meet a long-felt want among a particular class of patients. He believed that if the existence of these special beds were better known there would be more applications for them than could be dealt with.

The report and accounts were then adopted, and the usual votes of thanks were passed.

